# Prior beta-blocker treatment improves outcomes in out-of-hospital cardiac arrest patients with non-shockable rhythms

**DOI:** 10.1038/s41598-021-96070-8

**Published:** 2021-08-19

**Authors:** Hui-Chun Huang, Ping-Hsun Yu, Min-Shan Tsai, Kuo-Liong Chien, Wen-Jone Chen, Chien-Hua Huang

**Affiliations:** 1 Division of Cardiology, Department of Internal Medicine, National Taiwan University Hospital, National Taiwan University College of Medicine, Taipei, Taiwan; 2 Graduate Institute of Epidemiology and Preventive Medicine, College of Public Health, National Taiwan University, Taipei, Taiwan; 3 Department of Emergency Medicine, Taipei Hospital, Ministry of Health and Welfare, New Taipei City, Taiwan; 4 Department of Emergency Medicine, College of Medicine, National Taiwan University, No. 7 Chung-Shan South Rd., Taipei, 100 Taiwan

**Keywords:** Outcomes research, Interventional cardiology

## Abstract

The prognosis of out of cardiac arrest is poor and most cardiac arrest patients suffered from the non-shockable rhythm especially in patients without pre-existing cardiovascular diseases and medication prescription. Beta-blocker (ß-blocker) therapy has been shown to improve outcomes in cardiovascular diseases such as heart failure, ischemia related cardiac, and brain injuries. Therefore, we investigated whether prior ß-blockers use was associated with reduced mortality in patients with cardiac arrest and non-shockable rhythm. We conducted a population-based retrospective cohort study using multivariate propensity score–based regression to control for differences among patients with cardiac arrest. A total of 104,568 adult patients suffering a non-traumatic and non-shockable rhythm cardiac arrest between 2005 and 2011 were identified. ß-blocker prescription at least 30 days prior to the cardiac arrest event was defines as the ß-blockers group. We chose 12.5 mg carvedilol as the cut-off value and defined greater or equal to carvedilol 12.5 mg per day and its equivalent dose as high-dose group. After multivariate propensity score–based logistic regression analysis, patients with prior ß-blockers use were associated with better 1-year survival [adjusted odds ratio (OR), 1.15, 95% confidence interval (CI) 1.01–1.30; *P* = 0.031]. Compared to non-ß-blocker use group and prior low-dose ß-blockers use group, prior high-dose ß-blockers use group was associated with higher mechanical ventilator wean success rate (adjusted OR 1.19, 95% CI 1.01–1.41, *P* = 0.042). In conclusion, prior high dose ß-blockers use was associated with a better 1-year survival and higher weaning rate in patients with non-shockable cardiac arrest.

## Introduction

More than 356,000 people experience out-of-hospital cardiac arrest (OHCA) annually in the U.S., and almost 90% cases are considered fatal^1^. Pharmacotherapy can prevent disease progression; and effective resuscitation and rapid care for OHCA can lead to better outcomes; however, the long-term survival remains poor. Cardiac arrest with non-shockable rhythm is more prevalent at present and is associated with higher risk of mortality than shockable rhythm^2^. Also, people suffering cardiac arrest with non-shockable rhythm have less predisposing cardiac disease^3^.

Patients might experience complex pathophysiological processes such as brain injury, cardiac dysfunction, global ischemia–reperfusion injuries and a sepsis-like syndrome with cytokine storms through the duration of cardiac arrest^4^. Beta blockers (β-blocker) are drugs widely used to treat patients with cardiac arrhythmia, heart failure, and coronary artery disease^5^. β-blockers use is associated with better prognosis in patients with heart failure, myocardial infarction, cardiac arrhythmia, and acute stroke^6–8^. Whether prior β-blockers use improves survival in patients with cardiac arrest and non-shockable rhythm remains unclear. Therefore, this study aimed to determine if the prior use of β-blocker is associated with better survival and improved clinical and functional outcomes in OHCA patients with non-traumatic and non-shockable rhythms.

## Materials and methods

### Data sources

The mandatory health insurance in Taiwan, which now covers more than 99% of the Taiwanese population (23.78 million people), began in 1995 through the National Health Insurance (NHI) program^9^. All medical institutions contracted with the NHI program are required to submit standard computerized claims documents for medical expenses. Thus, all the details of claims, patients’ demographics, and medical care procedures are stored in the National Health Insurance Research Database (NHIRD). This retrospective and observational study was based on a nationwide population cohort. The study used electronic health records data as well as unstructured text found in clinical notes and reports accumulated between 2005 and 2011. The codes of the International Classification of Diseases, Ninth Revision, Clinical Modification (ICD-9-CM) were used for disease diagnoses. To conform to the rules of data privacy, personal information was encrypted, and was transformed to a de-identified manner. The study protocol was approved by the National Taiwan University Hospital Research Ethics Committee (study no. 201811086RINA) and waived the requirement of informed consent. All study methods were carried out in accordance with relevant guidelines and regulations.

### Patient selection and follow-up

**Figure 1:**
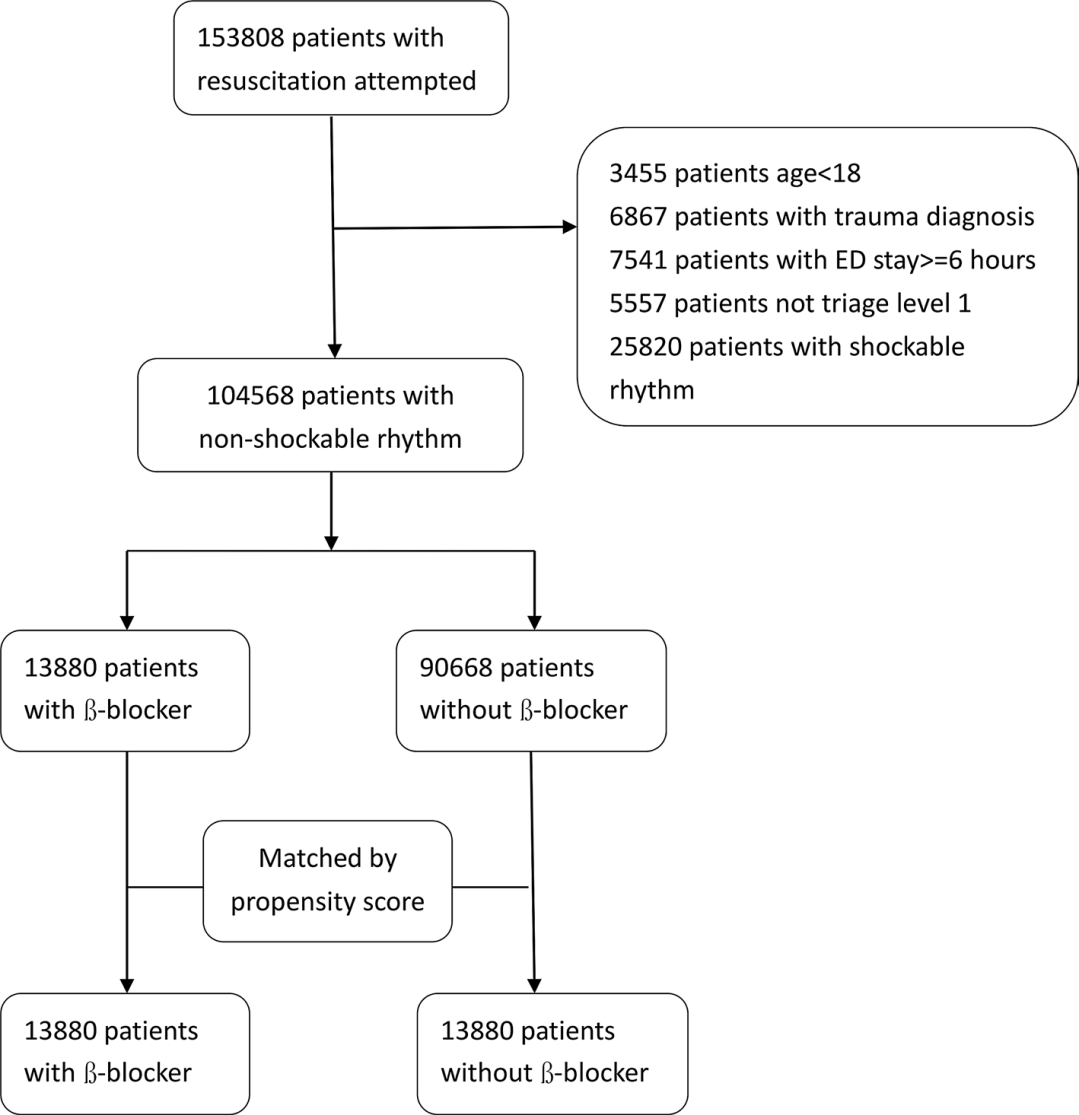
Flow chart of patient selection.

The study population included all patients receiving cardiopulmonary resuscitation (CPR) at the emergency room. These patients would be coded with a procedure code “47029c”. To appropriately select post-cardiac arrest patients, patients with (1) non-level 1 triage, (2) trauma-related event experience, (3) more than 6 h stay in emergency room, (4) shockable rhythm, and (5) < 18 years old were excluded from the study (Fig. 1). Patients presenting to emergency room in Taiwan are allocated a triage category based on the time in which they need medical attention. People who need to have treatment immediately or within two minutes are categorized as Level 1(resuscitation) and they are in an immediately life-threatening condition. Reportedly, only 3.9% of patients were assigned into levels 1^10^. Previous study revealed that the hospital mortality was associated with the ER boarding time. Patients stayed longer than 6 h in emergency room (coded in our National Health Insurance Research Database (NHIRD) were excluded to minimize the confounding effects of inadequate post-cardiac arrest care^11^.

Patients were followed-up from the index date of cardiac arrest until 1-year survival or death. The “β-blocker group” is composed of cardiac arrest patients using β-blockers continuously for at least 30 days before the index date. Patients without β-blocker use in the 30 days before the cardiac arrest event were included in the control group or “non-β-blocker group.” To investigate the dose effect of β-blockers on the 1-year survival in these patients, they were categorized to high-dose if the mean daily dose was greater or equal to carvedilol 12.5 mg or its equivalent dose. The Taiwan National Health Research Institute definitions of urbanization, hospital classification, and qualifications of medical center criteria have been previously described^12,13^ (Fig. 1).

### Outcome measures

The primary end point was 1-year survival. Additional clinical outcomes included survival to ICU admission and survival to discharge. The functional outcomes measured were ventilator wean success rate at 1 month.

### Statistical analysis

Descriptive statistics were computed for the categorical and continuous variables. We compared baseline characteristics of the 2 groups using the χ^2^ test for categorical variables and the *t* test for continuous variables. In addition to sex and age at index date, comorbidities at baseline and 1-year period were extracted from both outpatient and inpatient records and classified according to the Deyo-Charlson Comorbidity Index (Deyo-CCI)^14^.

Patients in the β-blocker and non-β-blocker groups were then matched by Propensity score (PS) at a 1:1 ratio sampling without replacement to reduce selection bias between the groups. Table 1 showed the results of logistic regression for PS estimation. Kaplan–Meier survival curves were analyzed using the stratified log-rank test to evaluate the β-blockers beneficial effect on 1-year survival. If the imbalance existed after PS matching, multivariate logistic regression analysis after PS matching (double-adjustment) could be performed to remove confounding^15,16^. The independent effects of prior β-blocker use on 1-year survival were analyzed by multivariate PS-based logistic regression analysis. The model was adjusted by potential confounders such as age, sex, drug use, CCI scores, underlying comorbidities like HTN, diabetes, coronary artery disease, congestive heart failure, atrial fibrillation, CKD, malignancy, COPD and asthma, urbanization level, the event year, resuscitation in medical center, hypothermia therapy, and achieved of coronary angiography. Subgroup analyses of 1-year survival were used for β-blockers and non-β-blockers groups based on clinical characteristics such as age, gender, CCI scores, diabetes mellitus, hypertension, dyslipidemia, CAD, CHF, atrial fibrillation, CKD, COPD and asthma. Differing criteria in sensitivity analysis were used to validate regression model findings. All analyses were performed with SAS software, version 9.4 (SAS Institute, Inc., Cary, North Carolina). All p-values reported were 2-tailed, and the significant level was set at < 0.05.

**Table 1:** Baseline characteristics, management and outcomes of patient with/without ß-blocker use before matching.

	Before Propensity score matching			
	All N = 104,568	ß-blocker N = 13,880	Non-ß-blocker N = 90,688	*p* value
Age, (SD), (years)	68.3 (17.8)	69.7 (14.3)	68.1 (18.3)	0.31
Male, (%)	65,061 (62.2)	7513 (54.1)	57,548 (63.5)	< 0.0001
**Medication**				
Antiplatelet agents	32,011 (30.6)	7069 (50.9)	24,942 (27.5)	< 0.0001
Angiotensin converting enzyme inhibitors	18,821 (18.0)	4057 (29.2)	14,764 (16.3)	< 0.0001
Angiotensin receptor blockers	22,905 (21.9)	5569 (40.1)	17,336 (19.1)	< 0.0001
Charlson comorbidity index,(SD)	2.3 (2.2)	2.9 (2.2)	2.2 (2.2)	< 0.0001
**Primary cause of the cardiac arrest**				
Asphyxia	2821 (2.7)	345 (2.5)	2476 (2.7)	0.10
Drowning	324 (0.3)	33 (0.2)	291 (0.3)	0.11
Electrocution	143 (0.2)	21 (1.5)	122 (1.3)	0.41
Drug overdose	44 (0.04)	1 (0.01)	43 (0.05)	0.025
**Medical**				
Cardiac	9048 (8.7)	1580 (11.4)	7648 (8.2)	< 0.001
Other medical cause				
Asthma	2227 (2.1)	164 (1.2)	2063 (2.3)	< 0.0001
Renal disease	3445 (3.3)	713 (5.1)	2732 (3.0)	< 0.0001
GI bleeding	1366 (1.3)	187 (1.4)	1179 (1.3)	0.6303
Unknown or no obvious	85,150 (81.4)	10,836 (78.1)	74,134 (81.7)	< 0.001
**Comorbidities**				
Diabetes mellitus	30,066 (28.8)	5861 (42.2)	24,205 (26.7)	< 0.0001
Hypertension	50,859 (48.6)	10,688 (77.0)	40,171 (44.3)	< 0.0001
Coronary artery disease	20,236 (19.4)	4922 (35.5)	15,314 (16.9)	< 0.0001
Congestive heart failure	17,616 (16.9)	3710 (26.7)	13,906 (15.3)	< 0.0001
Atrial fibrillation	4334 (4.1 )	878 (6.3 )	3456 (3.8 )	< 0.0001
Chronic kidney disease	10,904 (10.4)	2330 (16.8)	8574 (9.5 )	< 0.0001
Malignancy	12,644 (12.1)	1467 (10.6)	11,177 (12.3)	< 0.0001
Chronic obstructive pulmonary disease	27,863 (26.7)	3146 (22.7)	24,717 (27.3)	< 0.0001
Asthma	10,257 (9.8 )	1195 (8.6)	9062 (9.9 )	< 0.0001
**Urbanization level, n(%)**				0.21
1 (highest)	64,316 (61.5)	8623 (62.1)	55,693 (61.4)	
2	34,745 (33.2)	4559 (32.9)	30,186 (33.3)	
3	3931 (3.8 )	510 (3.8 )	3421 (3.8 )	
4 (lowest)	1576 (1.5 )	188 (1.5 )	1388 (2.2 )	
Resuscitation in medical center (%)	22,947 (21.9)	3280 (23.6)	19,667 (21.7)	< 0.0001
**Year of resuscitation**				< 0.0001
2005	13,902 (13.3)	1669 (12.0)	12,233 (13.5)	
2006	13,843 (13.2	1744 (12.6)	12,099 (13.3)	
2007	14,916 (14.3)	1907 (13.7)	13,009 (14.3)	
2008	15,624 (14.9)	2094 (15.1)	13,530 (14.9)	
2009	15,287 (14.6)	2100 (15.1)	13,187 (14.5)	
2010	15,264 (14.6)	2118 (15.3)	13,146 (14.5)	
2011	15,732 (15.0)	2248 (16.2)	13,484 (14.9)	
**Managements at emergency room**				
Epinephrine (%)	99,934 (95.6)	13,300 (95.8)	86,634 (95.5)	0.1211
Epinephrine dose(SD),(mg)	10.0 (19.1)	10.1 (8.0 )	9.9 (20.3)	0.0012
Vasopressin (%)	2309 (2.2 )	317 (2.3 )	1992 (2.2 )	0.5148
**Managements after ICU admission**				
Coronary angiography (%)	362 (0.4 )	87 (0.6 )	275 (0.3 )	< 0.0001
Hypothermia (%)	719 (0.7 )	127 (0.9 )	592 (0.7 )	0.0008
Ventilator (%)	14,260 (13.6)	2403 (17.3)	11,857 (13.1)	< 0.0001
**Outcomes**				
Survival to ICU admission	23,588 (22.6)	3571 (25.7)	20,017 (22.1)	< 0.0001
Survival to discharge (%)	14,243 (13.6)	2148 (15.5)	12,095 (13.3)	< 0.0001
One-year survival (%)	3377 (3.2 )	586 (4.2 )	2791 (3.1 )	< 0.0001
Ventilator wean rate in 30 days (%)	2687 (2.6)	461 (3.3 )	2226 (2.5 )	< 0.0001

## Results

**Figure 2:**
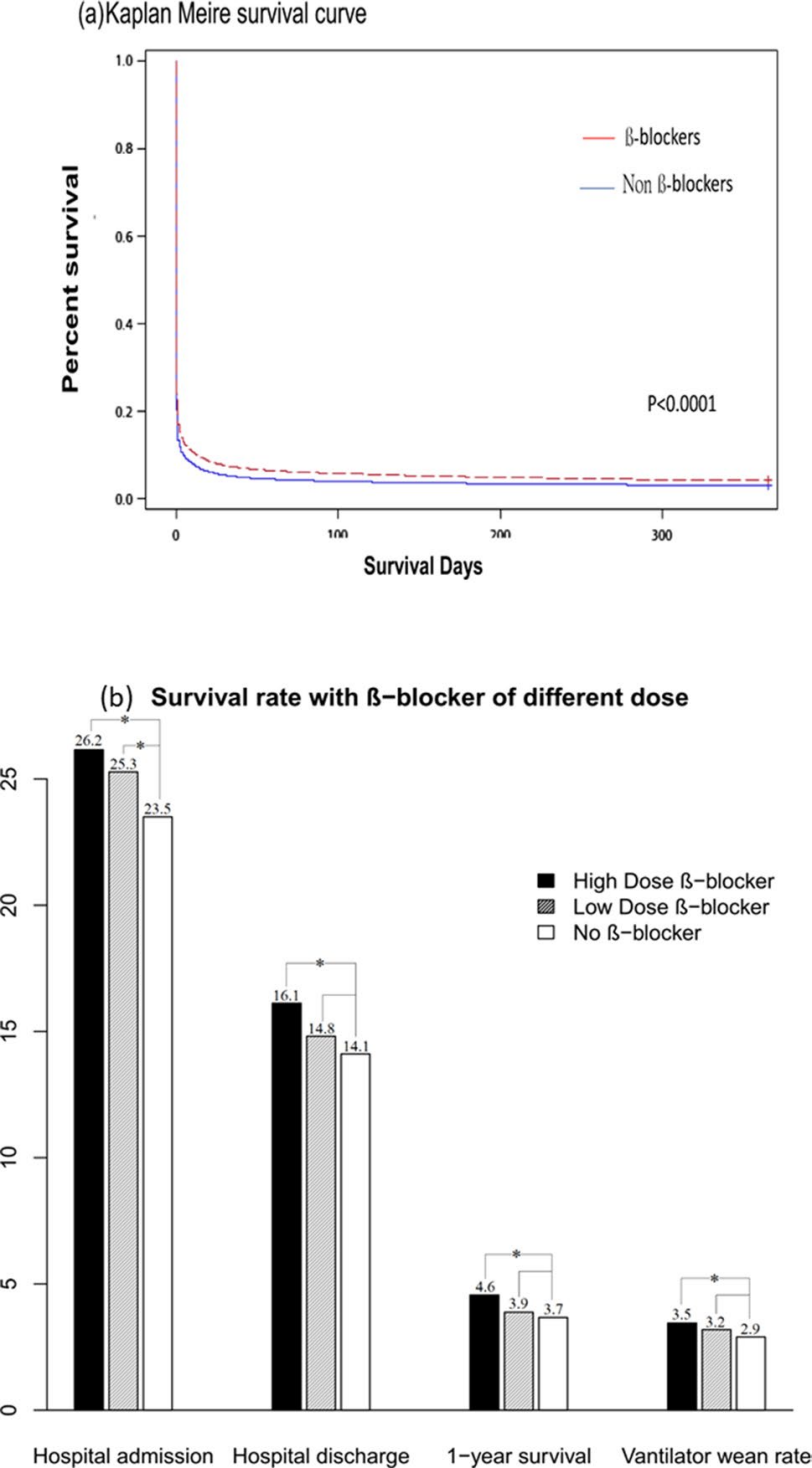
One-year survival rate for cardiac arrest people with or without prior ß-blockers use. (**a**) Kaplan–Meier one year survival curve in patients with/without prior ß-blockers use. (**b**) Multiple logistic regression analysis of (1) survival to hospital admission (2) survival to discharge (3) one year survival rate (4) weaning off ventilator rate among patients receiving high dose, low dose and no ß-blocker (*P < 0.05 by stratified log-rank test).

Overall, 104,568 patients were included in the analysis (Fig. 1), of which, 13,880 patients (13.3%) were β-blocker users. Patients in the β-blocker group had more comorbidities such as hypertension, diabetes malleus, coronary artery disease, congestive heart failure, and chronic kidney disease. Conversely, several patients in the control group had a history of malignancy, COPD, and asthma. The CCI score was higher and procedures for coronary angiography were performed more often in the β-blocker group. After PS matching, no significant differences in most comorbidities, gender, or year of resuscitation were noted between these groups except that patients in the non-β-blocker group were older, more hypertensive, had history of COPD, and less coronary artery disease. Our results showed that the survival to ICU admission (25.7% vs. 23.1%; *P* < 0.0001), survival to hospital discharge (15.5% vs. 13.7%; *P* < 0.0001), and 1-year survival (4.2% vs. 3.7%; *P* = 0.02) were better in the prior β-blocker use group after PS matching (Table 2). The Kaplan–Meier survival curve demonstrated better 1-year survival in the prior β-blockers use group (Fig. 2A, stratified log-rank test; *P* < 0.0001).

**Table 2:** Baseline characteristics, management and outcomes of patient with/without ß-blocker use after matching.

	After Propensity score matching		
	ß-blocker N = 13,880	Non-ß-blocker N = 13,880	*P* value
Age (SD), (years)	69.7 (14.3)	70.2 (15.8)	< 0.0001
Male (%)	7513 (54.1)	7605 (54.8)	0.27
**Medication**			
Antiplatelet agents	7069 (50.9)	6995 (50.4)	0.38
Angiotensin converting enzyme inhibitors	4057 (29.2)	4084 (29.4)	0.73
Angiotensin receptor blockers	5569 (40.1)	5527 (39.8)	0.62
Charlson comorbidity index, (SD)	2.9 (2.2)	2.9 (2.3)	0.84
**Comorbidities**			
Diabetes mellitus	5861 (42.2)	5832 (42.0)	0.73
Hypertension	10,688 (77.0)	11,011 (79.3)	< 0.0001
Coronary artery disease	4922 (35.5)	4744 (34.2)	0.025
Congestive heart failure	3710 (26.7)	3593 (25.9)	0.11
Atrial fibrillation	878 (6.3)	868 (6.3)	0.82
Chronic kidney disease	2330 (16.8)	2235 (16.1)	0.12
Malignancy	1467 (10.6)	1442 (10.4)	0.63
Chronic obstructive pulmonary disease	3146 (22.7)	3359 (24.2)	0.002
Asthma	1195 (8.6 )	1275 (9.2)	0.09
**Urbanization level, n(%)**			0.72
1 (highest)	8623 (62.1)	8618 (62.1)	
2	4559 (32.9)	4596 (33.1)	
3	510 (3.7)	476 (3.4)	
4 (lowest)	188 (1.4)	190 (1.4)	
Resuscitation in medical center (%)	3280 (23.6)	3289 (23.7)	0.91
**Year of resuscitation**			0.90
2005	1669 (12.0)	1632 (11.8)	
2006	1744 (12.6)	1777 (12.8)	
2007	1907 (13.7)	1952 (14.1)	
2008	2094 (15.1)	2093 (15.1)	
2009	2100 (15.1)	2128 (15.3)	
2010	2118 (15.3)	2067 (14.9)	
2011	2248 (16.2)	2231 (16.1)	
**Managements at emergency room**			
Epinephrine (%)	13,300 (95.8)	13,289 (95.7)	0.76
Epinephrine dose(SD),(mg)	10.1 (8.0)	9.9 (47.3)	0.006
Vasopressin (%)	317 (2.3)	329 (2.4)	0.66
**Managements after ICU admission**			
Coronary angiography (%)	87 (0.6)	75 (0.5)	0.38
Hypothermia (%)	127 (0.9)	86 (0.6)	0.005
**Outcomes**			
Survival to ICU admission	3571 (25.7)	3210 (23.1)	< 0.0001
Survival to discharge (%)	2148 (15.5)	1899 (13.7)	< 0.0001
One-year survival (%)	586 (4.2)	510 (3.7)	< 0.020
Ventilator wean rate in 30 days (%)	461 (3.3)	409 (3.0)	0.08

Under the multivariate PS-based logistic regression analysis, patients with prior ß-blockers use were associated with better 1-year survival under the (OR, 1.15, 95% CI 1.01–1.30; *P* = 0.031) after adjusting age, gender, medications use such as antiplatelet agents and ACEI/ARB, CCI scores, clinical covariates such as diabetes mellitus, hypertension, dyslipidemia, CAD, CHF, atrial fibrillation, CKD, COPD, urbanization level, the event year and the management of cardiac arrest(Table 3). A beneficial outcome of 1-year survival was observed for patients who were younger, were females, had a history of COPD, and had higher CCI scores (Table 3). Cardiac resuscitation that was performed at the medical center and required a lower amount of adrenaline (epinephrine) was associated with better chances of 1-year survival. In comparison to those who did not undergo coronary angiography, patients who underwent coronary angiography during hospitalization also had better chances of 1-year survival.

**Table 3:** Univariate and multivariate logistic analysis for factors related to one-year survival outcomes in after propensity score-1:1 match groups.

	Univariate		Multivariate	
	OR (95% CI)	*p* value	OR (95% CI)	*P* value
Age (per year)	0.982 (0.978–0.985)	< 0.0001	0.968 (0.964–0.973)	< 0.0001
Male	0.790 (0.700–0.892)	< 0.0001	0.736 (0.647–0.837)	< 0.0001
**Medication**				
Anti-platelet agents	1.358 (1.201–1.534)	< 0.0001	1.141 (0.985–1.323)	0.0796
Angiotensin-converting enzyme inhibitors	1.337 (1.178–1.517)	< 0.0001		
Angiotensin receptor blockers	1.655 (1.466–1.868)	< 0.0001	1.420 (1.237–1.629)	< 0.0001
Beta-blocker	1.156 (1.024–1.304)	0.0193	1.148 (1.012–1.302)	0.0315
CCI index	1.099 (1.072–1.126)	< 0.0001	1.083 (1.036–1.133)	0.0005
**Comorbidities**				
Diabetes mellitus	1.300 (1.152–1.467)	< 0.0001	0.924 (0.785–1.088)	0.3423
Hypertension	1.091 (0.939–1.267)	0.2540	1.164 (0.978–1.384)	0.0867
Coronary artery disease	1.460 (1.292–1.650)	< 0.0001	1.233 (1.067–1.425)	0.0045
Congestive heart failure	1.494 (1.315–1.698)	< 0.0001	1.132 (0.972–1.319)	0.1119
Atrial fibrillation	1.232 (0.980–1.549)	0.0744	1.176 (0.919–1.504)	0.1982
Chronic kidney disease	1.489 (1.287–1.723)	< 0.0001	0.990 (0.820–1.195)	0.9129
Malignancy	0.627 (0.495–0.794)	0.0001	0.526 (0.399–0.694)	< 0.0001
Chronic obstructive pulmonary disease	1.449 (1.270–1.653)	< 0.0001	1.534 (1.318–1.785)	< 0.0001
Asthma	1.451 (1.205–1.747)	< 0.0001		
**Urbanization level**				
1 (highest)	0.926 (0.558–1.537)	0.3486	1.230 (0.695–2.176)	0.4775
2	0.972 (0.583–1.620)	0.1530	1.210 (0.681–2.153)	0.5156
3	0.589 (0.311–1.115)	0.0250	0.825 (0.412–1.649)	0.5855
4 (lowest)	1		1	
Resuscitation in medical center	1.354 (1.185–1.574)	< 0.0001	1.320 (1.145–1.520)	0.0001
**Year of events**				
2005	1			
2006	1.152 (0.898–1.478)	0.6411		
2007	1.123 (0.879–1.436)	0.8796		
2008	1.060 (0.831–1.353)	0.5385		
2009	1.351 (1.071–1.706)	0.0043		
2010	0.998 (0.779–1.277)	0.1656		
2011	1.126 (0.888–1.428)	0.8452		
**Mangement**				
Epinephrine dose (per mg)	0.873 (0.861–0.886)	< 0.0001	0.883 (0.871–0.896)	< 0.0001
Vasopressin	0.693 (0.432–1.112)	0.1283		
Coronary angiography	28.29 (20.66–38.75)	< 0.0001	18.56 (12.92–26.66)	< 0.0001
Hypothermia	6.557 (4.679–9.189)	< 0.0001		

The mean daily dose in both groups is listed in Table 4. After using Carvedilol 12.5 mg and its equivalent dose as cutoff value, the mean daily dose greater or equal to Carvedilol 12.5 mg (or its equivalent dose) was defined as high-dose group. After multivariate PS-based logistic regression analysis, both patients in the high-dose (n = 7021) and low-dose (n = 6859) prior β-blockers use groups had greater odds of survival to hospital admission (adjusted OR: 1.16, 95% CI 1.09–1.25, *P* < 0.0001 and adjusted OR: 1.08, 95% CI 1.01–1.16, *P* = 0.0216, respectively ) and survival to discharge (adjusted OR: 1.18, 95% CI 1.09–1.28, *P* < 0.0001, and adjusted OR: 1.04, 95% CI 0.95–1.13, *P* = 0.41, respectively) than those in the control group. In addition, the high-dose prior β-blockers use group had better chance of 1-year survival (adjusted OR: 1.27, 95% CI 1.09–1.47, *P* = 0.0021) and higher ventilator wean rate (adjusted OR: 1.19, 95% CI 1.01–1.411, *P* = 0.0423) than the control group after multivariate PS-based logistic regression analysis (Fig. 2B).

**Table 4:** The mean daily dose use of ß-blocker in both high dose and low dose group after propensity score matching.

ß-blocker	N	Mean daily dose (mg)	Median daily dose (mg)	High dose group	Low dose group
Propranolol	3908	19.7	15.1	N = 461 45.5 (33.8)	N = 3447 16.2 (8.2)
Carvedilol	3396	15.2	10.4	N = 1487 25.6 (13.6)	N = 1909 7.1 (2.6)
Bisoprolol	3209	3.4	3.1	N = 1964 4.4 (1.8)	N = 1245 1.8 (0.4)
Atenolol	2369	63.2	54.9	N = 2238 65.6 (33.3)	N = 131 22.3 (1.4)
Labetalol	899	303.3	279.1	N = 838 320.0 (176.0)	N = 61 74.5 (13.0)
Metoprolol	319	69.1	59.1	N = 192 91.7 (41.7)	N = 127 35.0 (7.9)
Acebutolol	184	316.8	274.3	N = 177 326.0 (188.6)	N = 7 84.2 (10.5)
carteolol	48	7.5	7.1	N = 29 10.0 (3.2)	N = 19 3.8 (0.6)
Nadolo	23	60.8	51.5	N = 14 81.5 (41.5)	N = 9 28.6 (3.5)
Sotalol	11	119.8	117.9	N = 8 145.3 (49.3)	N = 3 51.8 (19.4)
Pindolol	10	6.9	5.1	N = 5 10.1 (2.8)	N = 5 3.7 (1.0)

we defined the mean daily dose greater or equal to Carvedilol 12.5 mg qd and its equivalent dose as high dose group.

**Figure 3:**
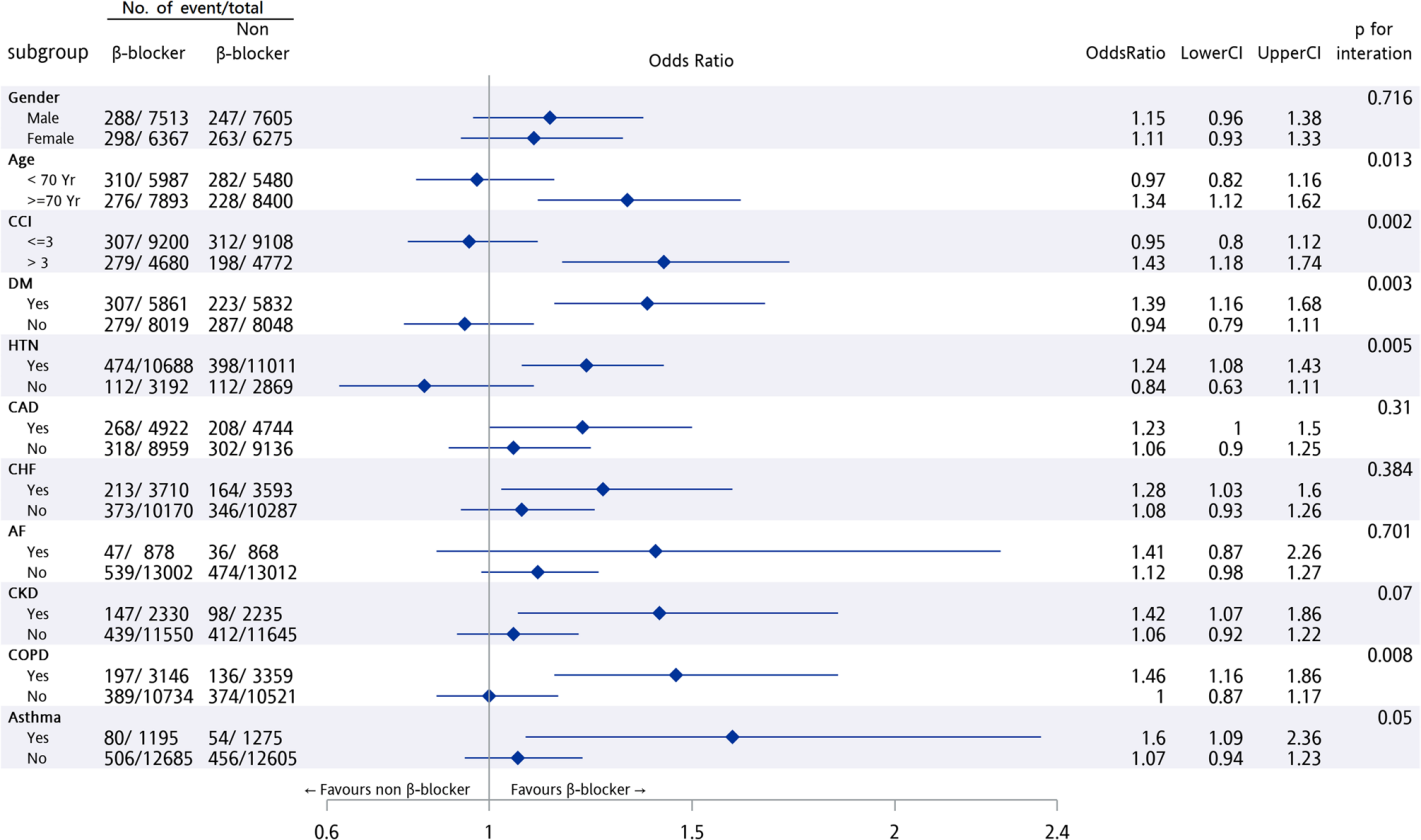
Subgroup analysis among prior ß-blockers use before cardiac arrest, clinical characteristics, and one-year survival (*P < 0.05).

Also, subgroup analysis was performed in patients with prior β-blockers use, we found that compared to patents with prior β-blockers use and younger than 70 years old, patients with prior β-blocker use and more than 70 years old are associated with a better 1-year survival. (adjusted OR: 1.12 vs. 1.62, P-value of interaction = 0.013). Similar finding was also found in patients with the history of diabetes (adjusted OR: 1.16 vs. 1.68, P-value of interaction = 0.003), hypertension (adjusted OR: 1.08 vs. 1.43, P-value of interaction = 0.005), and COPD (adjusted OR: 1.16 vs. 1.86, P-value of interaction = 0.008), and with a CCI score > 3 (OR: 1.18 vs. 1.74 P-value of interaction = 0.002). The remaining subgroups were not significantly different on the outcome of 1-year survival. (Fig. 3). Further large multicenter prospective studies are required to verify the better 1-year survival outcome of prior β-blocker use in patients > 70 years old, with history of HTN, diabetes, COPD or CCI score > 3.

As shown in Table 5, sensitivity analysis excluded patients with comorbidities such as COPD, asthma or malignancy, prior to cardiac arrest. Patients receiving no epinephrine or only 1 dose (due to rhythm conversion, without resorting to anti-arrhythmic agents) were also excluded in the sensitivity analysis. The relationships between high- or low-dose β-blocker treatment and 1-year survival, as well as survival to ICU admission and survival to hospital discharge, remained consistent.

**Table 5:** Sensitivity analysis of the logistic regression model for one-year survival outcomes in cardiac arrest patients with non-shockable rhythm.

	Adjusted odds ratio	95% CI	*p* value
**Primary analysis**			
All ß-blockers	1.140	1.032–1.258	0.0097
High dose ß-blockers	1.273	1.119–1.447	0.0002
Low dose ß-blockers	1.017	0.888–1.164	0.81
**Excluding patients with COPD/asthma**			
All ß-blockers	1.038	0.920–1.172	0.54
High dose ß-blockers	1.151	0.985–1.345	0.040
Low dose ß-blockers	0.930	0.787–1.098	0.39
**Excluding patients with pre-existing malignancy**			
All ß-blockers	1.120	1.010–1.242	0.031
High dose ß-blockers	1.254	1.098–1.433	0.0009
Low dose ß-blockers	0.995	0.863–1.146	0.94
**Excluding patients receiving less than or equal to 1 mg epinephrine N = 96,852**			
All ß-blockers	1.196	1.065–1.343	0.0026
High dose ß-blockers	1.338	1.151–1.555	0.0001
Low dose ß-blockers	1.065	0.909–1.249	0.43

## Discussion

Our nationwide cohort study showed that a greater odd of better 1-year survival and higher weaning rate of ventilator were observed in the prior high-dose β-blocker use group under the multivariate PS-matched regression analysis method. Reportedly, the chance of 1-year survival after cardiac arrest was higher in patients with favorable neurologic outcomes at hospital discharge^17^. From our analysis of ventilator wean rates, pre-β-blocker use not only improved the long-term clinical outcome but also improved the immediate functional outcomes after cardiac arrest. Better outcomes of previous ß-blockers use in patients with non-shockable cardiac arrest were unchanged even after sensitivity analysis.

The Charlson Comorbidity Index (CCI) score is a measure of the burden of disease arising from multiple comorbidities and a higher CCI score was associated with greater mortality risk^18^. The existence of some comorbidity may trigger the initiation of appropriate therapy to reduce that mortality risk. High CCI scores might be associated with more intensive treatment, and this could lead to inadequate prediction of 1-year survival due to the presence of a treatment paradox^19^.

Cardiac arrest patients experience post cardiac arrest syndrome, which comprises anoxic brain injury, post cardiac arrest myocardial dysfunction, the global ischemic-reperfusion injuries, and responsible for mortality and morbidity in these patients^20^. Cessation of blood circulation leads to ischemia reperfusion injuries in major organs, including the brain and the heart. The ischemic event initiates a cascade of circulating inflammatory cytokines, resulting in excessive sympathoadrenal activation and endothelial damage. The pathological sympathetic activation after acute ischemic stroke has been repeatedly observed in acute stroke and is related to poorer outcomes^21^.β-blockers use before ischemia-related brain injuries showed beneficial effects including a milder stroke severity and lower mortality^22^. In animal models, β-blockers administered before the induction of experimental ischemia lead to a reduction in infarct volume by 40%^23^. The high susceptibility to pneumonia caused by the sympathetic activation in post-stroke immunosuppression has been extensively discussed. Stroke treatment with β-blockers has been associated with reduced mortality^24^.

Several studies have shown the protective effect of β-blockers on ischemia-related cardiac injury^23,24^. The Cardiac Insufficiency Bisoprolol Study II (CIBIS-II) trial, the first efficacy trial investigating all-cause mortality in patients with chronic heart failure on β-blocker therapy, showed a 32% risk reduction in total mortality^25^. The use of pre-procedural β-blockers was associated with a lower risk of in-hospital cardiac death in patients with acute coronary syndrome undergoing percutaneous coronary intervention^26^. The perioperative cardioprotective effect made by β-blockers may be multifactorial. First, the negative inotropic and chronotropic effects made byβ-blockers decrease myocardial oxygen demand. Also, β-blockers could modulate myocardial inflammatory responses such as cytokine release and leukocyte recruitment^27^. Moreover, the myocardial metabolism was shift from fatty acid oxidation toward glucose uptake^28^. Therefore, it is reasonable to suggest that prior β-blockers use might mitigate the magnitude of post cardiac arrest syndrome, which results in improved clinical and functional outcomes and overall performance survival.

Prior systematic review of clinical trials about ß-blockers use in patients with heart failure has established higher dose ß-blocker does not ameliorate better outcomes^29^. Take CIBIS II study for example, the reduced mortality with bisoprolol was little different regardless of the dose level (high dose (10 mg/day), moderate dose (5 mg or 7.5 mg/day) and lowest dose (1.25, 2.5 or 3.75 mg/day)^30^. The incidence and severity of adverse drug effects are often dose related. ß-blockers can be effective in lower dose. In our high dose prior β-blockers use group, the mean daily dose was 4.4 mg (bisoprolol) or 25.6 mg (carvedilol). But the mean daily dose in low dose prior β-blockers use group was only 1.8 mg (bisoprolol) or 1.8 mg (carvedilol). The insufficient dose of ß-blocker use might explain the insignificant survival benefit in the low dose ß-blocker group.

The study has some limitations. Detailed neurologic outcomes were not available from the NHIRD. Instead, we evaluated the overall performance outcomes by ventilator wean success rate at 1 month. Also, resuscitation information at the area of occurrence was not accessible. However, the quality of resuscitation performance was not related to the prior use of β-blockers.

## Conclusion

In conclusion, patients with prior high-dose ß-blockers use before cardiac arrest are associated with better chance of 1-year survival and higher weaning rate in a propensity score-matched nationwide cohort study. Further large multicenter prospective studies are required to confirm whether prior ß-blockers use before cardiac arrest leads to improved clinical outcomes in cardiac arrest patients with non-shockable rhythm.

## Data Availability

The dataset used and analyzed during the current study are available from the corresponding author on reasonable request.
